# Resting Physiologic Dead Space as Predictor of Postoperative Pulmonary Complications After Robotic-Assisted Lung Resection: A Pilot Study

**DOI:** 10.3389/fphys.2022.803641

**Published:** 2022-07-18

**Authors:** Rohit Godbole, Sanford B. Church, Amir Abolhoda, Janos Porszasz, Catherine S. H. Sassoon

**Affiliations:** ^1^ Department of Medicine, Division of Pulmonary and Critical Care Medicine, University of California, Irvine, CA, United States; ^2^ Department of Medicine, Division of Pulmonary and Critical Care Medicine, VA Long Beach Healthcare System, Long Beach, CA, United States; ^3^ The Lundquist Institute for Biomedical Innovation at Harbor-UCLA Medical Center, Torrance, CA, United States

**Keywords:** physiologic dead space, postoperative predictor, pulmonary complications, robotic-assisted lung resection, lung cancer

## Abstract

Lung resection surgery carries significant risks of postoperative pulmonary complications (PPC). Cardiopulmonary exercise testing (CPET) is performed to predict risk of PPC in patients with severely reduced predicted postoperative forced expiratory volume in one second (FEV1) and diffusion of carbon monoxide (DLCO). Recently, resting end-tidal partial pressure of carbon dioxide (PETCO_2_) has been shown as a good predictor for increased risk of PPC. However, breath-breath breathing pattern significantly affects PETCO_2_. Resting physiologic dead space (VD), and physiologic dead space to tidal volume ratio (VD/VT), may be a better predictor of PPC than PETCO_2_. The objective of this study was to prospectively determine the utility of resting measurements of VD and VD/VT in predicting PPC in patients who underwent robotic-assisted lung resection for suspected or biopsy-proven lung malignancy. Thirty-five consecutive patients were included in the study. Patients underwent preoperative pulmonary function testing, symptom-limited CPET, and a 6-min walk test. In the first 2 min prior to the exercise portion of the CPET, we obtained resting VT, minute ventilation ( $\dot{V}$ E), VD (less instrument dead space), VD/VT, PETCO_2_, and arterial blood gases. PPC within 90 days were recorded. Fourteen (40%) patients had one or more PPC. Patients with PPC had significantly elevated resting VD compared to those without (0.318 ± 0.028 L vs. 0.230 ± 0.017 L (± SE), *p* < 0.006), and a trend toward increased VD/VT (0.35 ± 0.02 vs. 0.31 ± 0.02, *p* = 0.051). Area under the receiver operating characteristic (ROC) for VD was 0.81 (*p* < 0.002), VD/VT was 0.68 (*p* = 0.077), and PETCO_2_ was 0.52 (*p* = 0.840). Peak $\dot{V}$ O_2_, $\dot{V}$ E/ $\dot{V}$ CO_2_ slope, pulmonary function tests, 6-min walk distance and arterial blood gases were similar between the two groups. Intensive care unit and total hospital length of stay was significantly longer in those with PPC. In conclusion, preoperative resting VD was significantly elevated in patients with PPC. The observed increase in resting VD may be a potentially useful predictor of PPC in patients undergoing robotic-assisted lung resection surgery for suspected or biopsy-proven lung malignancy. A large prospective study is needed for confirmation.

## Introduction

Preoperative evaluation of lung function is the standard of care to estimate the risks of postoperative pulmonary complications (PPC) following lung resection for lung nodules, either biopsy-proven, or suspicious for cancer. Lung resection surgery carries significant risks, including postoperative respiratory failure, pneumonia, and atelectasis, resulting in prolonged hospital length of stay and mortality. Preoperatively, cardiopulmonary exercise testing (CPET), stair climb or shuttle-walk tests, as well as forced expiratory volume in 1 s (FEV_1_), diffusion capacity for carbon monoxide (DLCO) have been utilized to assess patients’ risks of PPC. If there is no increased cardiac risk for lung resection surgery, but severely reduced postoperative predicted FEV_1_ and/or DLCO, or poor performance of stair climb or shuttle-walk test, current guidelines recommend CPET (Brunelli et al., 2013). With CPET, subjects who underwent lobectomy or pneumonectomy, maximum oxygen utilization or peak oxygen uptake (Peak $\dot{V}$ O_2_) has been shown as a good predictor of morbidity and mortality (Brunelli et al., 2009). Subsequently, the same group of investigators reported the advantage of minute ventilation to carbon dioxide production ( $\dot{V}$ E/ $\dot{V}$ CO_2_) slope as predictor of PPC risks independent of Peak $\dot{V}$ O_2_ (Brunelli et al., 2012). Patients with $\dot{V}$ E/ $\dot{V}$ CO_2_ slope greater than 35 had high morbidity and mortality.

In healthy individuals, $\dot{V}$ E/ $\dot{V}$ CO_2_ ratio decreases during exercise with increasing workload (Wasserman et al., 1967). The ratio increases when $\dot{V}$ E is greater than $\dot{V}$ CO_2_ in response to metabolic acidosis. $\dot{V}$ E/ $\dot{V}$ CO_2_ is also dependent on physiologic dead space to tidal volume ratio (VD/VT) (Roman et al., 2013). According to balance of masses (Whipp, 2006), this can be demonstrated from the relationship between arterial partial pressure of carbon dioxide (PaCO_2_), $\dot{V}$ CO_2_, and $\dot{V}$ E/ $\dot{V}$ CO_2_ in which:

$$\dot{V}E/\dot{V}CO_{2}=\frac{k}{\left[PaCO_{2}x\left(1-VD/VT\right)\right]}$$ (1)

where k is constant, equals to 863. From Eq. 1, as Roman et al. (2013) demonstrated, high resting $\dot{V}$ E/ $\dot{V}$ CO_2_ is associated with high VD/VT, low PaCO_2_, end-tidal PCO_2_ (PETCO_2_), or both.

In a recent retrospective study, Brat et al. (2016) demonstrated elevated $\dot{V}$ E/ $\dot{V}$ CO_2_ slope and reduced end-tidal PCO_2_ (PETCO_2_) at both peak exercise and rest in patients with PPC, with resting PETCO_2_ as the strongest independent predictor. However, PETCO_2_, is affected by regional ventilation-perfusion mismatch and breathing pattern (Lewis et al., 1994). Resting VD or VD/VT has not been explored as a predictor of PPC risk after lung resection. Whereas the safety of symptom-limited CPET has been well documented, there is a substantial number of patients who are unable or unwilling to perform CPET (Keteyian et al., 2009). In this regard, the addition of resting test(s) that can effectively predict risks of PPC after lung resection will be beneficial.

The primary objective of our study was to determine if resting VD or VD/VT could reliably predict the risk of PPC within 90 days of robotic-assisted lung resection surgery in patients with a suspicious or biopsy-proven lung malignancy. The secondary objective was to compare the utility of resting VD or VD/VT with resting PETCO_2_ as a predictor of PPC in our population. We hypothesized that elevated resting VD or VD/VT will be useful and a better predictor of PPC risk than PETCO_2_.

## Materials and Methods

### Participants

Seventy-four patients with suspected or biopsy-proven lung malignancy were referred for lobectomy or segmentectomy by the multi-disciplinary team for lung cancer management at the Veteran Affairs Healthcare System, Long Beach, from January 2018 to January 2019. Thirty-nine patients were excluded for the following reasons: 1) declined to consent for the study (*n* = 11), declined or unable to perform CPET (*n* = 22), declined lung resection (*n* = 4), or had lung resection at another institution (*n* = 2). We studied the remaining 35 patients consecutively. Prior to the study each patient signed a written informed consent. Patients had to meet the following criteria: age greater than 18 years, non-pregnant, and ability to perform pulmonary function tests, six-minute walk test (6MWT), and CPET with no contraindications (Datta et al., 2015).

### Study Design

This was a prospective observational study approved by the institutional review board of the Veteran Affairs Healthcare System, Long Beach. Patients underwent preoperative pulmonary function testing, 6MWT, and CPET.

Spirometry and breath-by-breath gas analysis during CPET was performed using Vmax Encore^TM^ System (Vyaire Medical, Irvine, CA, United States). CPET was performed on average 12 days prior to surgery on incremental cycle ergometer (VIAsprint 150P^TM^, AIM, Sylmar, CA, United States). All equipment was calibrated prior to every test. After allowing the patient to adapt to the breathing apparatus and seated quietly on the cycle ergometer for at least 15 min, CPET commenced. CPET involved 2 min of rest, followed by 2 min of unloaded pedaling, then continued with application of ramp-incremental work-rate profile (5–15 W/min) to the point of symptom limitation (Datta et al., 2015). During CPET an average of 10 s data points were displayed. No patients discontinued CPET for cardiac events. Single arterial blood sample was obtained from each patient proximate to the CPET 2-min rest period via radial artery puncture using standard technique, and immediately analyzed via blood gas analyzer (RapidPoint 400 Series, Bayer Healthcare Systems, Oxnard, CA). Samples were obtained at room temperature and corrected for body temperature of 37^°^C. From Eq. 1, utilizing resting $\dot{V}$ E/ $\dot{V}$ CO_2_ averaged over 2-min and PaCO_2_ obtained from arterial blood gases, resting VD and VD/VT were calculated. Instrument dead-space measured by water displacement method three times, and the average value amounted to 169 ml was subtracted from the calculated VD. $\dot{V}$ E/ $\dot{V}$ CO_2_ slope was calculated using linear regression with $\dot{V}$ E as the dependent, and $\dot{V}$ CO_2_ the independent variable with onset of workload as the initial point to the peak workload as the end point. Nadir $\dot{V}$ E/ $\dot{V}$ CO_2_ was calculated, when available, at the ventilatory compensation threshold and average over 30 s (Mezzani, 2017). Finally, the 6MWT was conducted following complete resolution of symptoms from CPET.

One board-certified cardiothoracic surgeon (AA) performed all lung resections using robotic-assisted surgery. The surgical team managed patients postoperatively with patients mobilized as soon as tolerated. PPCs were recorded after a thorough chart review and included events immediately after surgery up to 90 days post-surgery. The PPCs included 1) pneumonia defined as increased sputum production, leukocytosis, fever, positive sputum culture, and consolidation on chest x-ray, 2) respiratory failure requiring invasive or non-invasive mechanical ventilation, or 3) atelectasis requiring bronchoscopy.

### Statistical Analysis

For this pilot study, a sample size of 24 subjects was required as determined using a mean difference of 25% according to the morbidity of postoperative lung resection reported by Brunelli et al. (2012), standard deviation of 30%, power of 0.80, and alpha of 0.05. Patients were grouped into those with and without PPC. Continuous variables were expressed as mean and standard deviation or standard error of the mean as appropriate; and compared using the unpaired two-tailed Student’s t test. Categorical variables, or those that did not pass the normality test and/or equal variance test, were compared using the Mann-Whitney Rank Sum test, and median with interquartile range values are reported. Differences in proportion were evaluated using the Fisher exact test. Receiver operating characteristic (ROC) curves were evaluated for threshold values for variables of interest, i.e., VD, VD/VT and PETCO_2_. In ROC, sensitivity, the dependent variable is the ability of a test to correctly identify patients with PPC, while specificity is the ability of a test to correctly identify those without PPC. Ideally, the threshold value has a sensitivity proximate 1.0 and 1-specificity (the independent variable) proximate 0, or the area under the ROC curve is close to 1.0. When the area under the ROC is statistically significant, the threshold value was determined from the highest sum of sensitivity and specificity according to the Youden approach (Bewick et al., 2004). Data were analyzed using Sigmaplot v.14 software (Systat Software Inc., San Jose, CA, United States).

## Results

Postoperative pulmonary complications occurred in 14 (40%) out of 35 patients with one or more complications occurring in the same patient. There was no mortality. All patients underwent lobectomy, except one patient in each group, with and without PPC, had segmentectomy.

Table 1 shows the baseline characteristics of patients in both groups with and without PPC. Average age in both groups was 70 years, and all patients were male Veterans. Tobacco use did not differ between groups, but was somewhat higher in those with (40 packyears) than without (25 packyears) PPC. A similar trend was shown in the prevalence of COPD with 79% of those having PPC compared to 52% in those without PPC. The prebronchodilator spirometry, DLCO, and 6-min Walk distance were similar in both groups (Table 2).

**TABLE 1:** Subjects characteristics.

Characteristics	Subjects with post-operative pulmonary complications (*n* = 14)	Subjects without post-operative pulmonary complications (*n* = 21)	*p*
Age (years)	70.3 ± 1.8	70.7 ± 1.5	0.859
Male (%)	100	100	
Height (m)	1.78 ± 0.02	1.78 ± 0.01	0.753
Weight (Kg)	84.4 ± 4.1	82.0 ± 3.0	0.626
Tobacco Use (packyears) ^†^	40 (15, 80)	25 (1, 50)	0.181
COPD, n (%)	11 (78.6)	10 (52.4)	0.163
CHF, n (%)	2 (14.3)	2 (9.5)	1.00

Values are mean ± SE, † is median with 25 and 75 percentiles in parenthesis.

Definition of abbreviations: COPD, chronic obstructive pulmonary disease; CHF, congestive heart failure.

**TABLE 2:** Pulmonary function and six-minute walk tests.

Variables	Subjects with post-operative pulmonary complications (*n* = 14)	Subjects without post-operative pulmonary complications (*n* = 21)	*p*
FEV1/FVC (%)	65.8 ± 3.3	70.0 ± 2.9	0.352
FEV1 (L)	2.33 ± 0.22	2.73 ± 0.15	0.120
FEV1 (% predicted)	73.5 ± 7.2	85.1 ± 4.1	0.143
FVC (L)	3.65 ± 0.27	3.96 ± 0.19	0.342
FVC (% predicted)	83.4 ± 5.7	92.0 ± 3.1	0.166
DLCO (ml/min/mm Hg)	20.3 ± 2.0	22.1 ± 1.8	0.517
DLCO (% predicted)	77.6 ± 8.6	83.5 ± 6.9	0.598
6 MWT (m)	400.4 ± 21.5	408.0 ± 14.8	0.766

Values are mean ± SE.

Definition of abbreviations: FEV1, forced expiratory volume in 1 s; FVC, forced expiratory vital capacity; DLCO, diffusing capacity for carbon monoxide; 6 MWT, six-minute walk test.

Table 3 demonstrates the measured variables at rest and with exercise during CPET. At rest, the PPC group had significantly elevated VD, average was 0.318 L versus 0.230 L in those without PPC (*p* < 0.006). VD/VT tended to be greater (*p* = 0.051) in the PPC group, while PETCO_2_, other ventilatory variables, and arterial blood gases were not significantly different from those without PPC (Table 3). Resting hyperventilation was not observed during CPET as corroborated by the pH and PaCO_2_.

**TABLE 3:** Cardiopulmonary exercise test at rest and exercise.

At rest			
Variables	Subjects with post-operative pulmonary complications (*n* = 14)	Subjects without post-operative pulmonary complications (*n* = 21)	*p*
VT (L)	0.904 ± 0.071	0.764 ± 0.043	0.084
Frequency (breaths/min)	20.1 ± 1.4	19.7 ± 0.9	0.816
V^˙^E (L/min)	17.4 ± 1.1	14.9 ± 1.0	0.110
V^˙^CO_2_ (L/min)	0.382 ± 0.03	0.348 ± 0.02	0.376
V^˙^O_2_ (L/min)	0.470 ± 0.04	0.395 ± 0.03	0.097
Respiratory Quotient	0.82 ± 0.02	0.81 ± 0.01	0.735
V^˙^E/V^˙^CO_2_	47.0 ± 2.6	43.6 ± 1.6	0.250
VD (L)	0.318 ± 0.03	0.230 ± 0.02	0.006*
VD/VT	0.35 ± 0.02	0.31 ± 0.02	0.051
PETCO_2_ (mm Hg)	32.0 ± 1.4	31.5 ± 1.0	0.763
pH (unit)	7.43 ± 0.01	7.43 ± 0.01	0.666
PaCO_2_ (mm Hg)	37.9 ± 1.9	35.6 ± 1.3	0.312
PaO_2_ (mm Hg)	78.5 ± 3.3	86.0 ± 3.5	0.153
SaO_2_ (%)	92.7 ± 0.7	92.8 ± 1.3	0.977
PETCO_2_-PaCO_2_	5.9 ± 0.9	4.02 ± 0.7	0.134
**With Exercise**			
Peak V^˙^O_2_ (L/min)	1.646 ± 0.127	1.686 ± 0.090	0.792
Peak V^˙^O_2_ (% PRED)	77.9 ± 5.6	77.7 ± 3.8	0.969
Peak V^˙^O_2_ (L/min/Kg)	19.6 ± 1.3	20.2 ± 1.1	0.494
V^˙^O_2_ at LT (L/min)	1.29 ± 0.10	1.38 ± 0.07	0.446
V^˙^O_2_ at LT (% of Peak V^˙^O_2_)	78.9 ± 2.5	81.2 ± 1.9	0.334
Peak Power (watts/min)	84.0 ± 6.3	95.0 ± 5.9	0.222
V^˙^E/V^˙^CO_2_ slope	33.0 ± 2.3	31.2 ± 1.1	0.446
Nadir V^˙^E/V^˙^˙CO_2_^†^	34.3 ± 1.1	32.4 ± 1.4	0.333

Values are mean ± SE. *p < 0.05.

Definition of abbreviations: VT, tidal volume; V^˙^E, minute ventilation; V^˙^O_2_, oxygen consumption; V^˙^CO_2_, carbon dioxide production; PETCO_2_, end-tidal carbon dioxide; VD, physiologic dead space volume; VD/VT, physiologic dead space to tidal volume ratio; LT, lactate threshold. † Measured at ventilatory compensation threshold, subjects with post-operative pulmonary complications (*n* = 12); without post-operative pulmonary complications (*n* = 16). See text for further explanation.

With exercise, peak $\dot{V}$ O_2_, $\dot{V}$ O_2_ at lactate threshold, peak power, $\dot{V}$ E/ $\dot{V}$ CO_2_ slope, and nadir $\dot{V}$ E/ $\dot{V}$ CO_2_ were similar in both groups. Nadir $\dot{V}$ E/ $\dot{V}$ CO_2_ was estimated in only those who attained ventilator compensation threshold, 12 and 15 patients in the group with and without PPC, respectively.

Postoperative pneumonia and atelectasis requiring therapeutic bronchoscopy were the most common PPCs (Table 4). As expected, patients with PPCs had significantly extended ICU and hospital length of stay compared with the group without PPC.

**TABLE 4:** ICU, hospital length of stay, and types of post-surgical complications.

	Subjects with post-operative pulmonary complications (*n* = 14)	Subjects without post-operative pulmonary complications (*n* = 21)	*p*
Pneumonia, n (%)	8 (57.1)	0 (0.0)	< 0.001
Atelectasis Requiring Bronchoscopy, n (%)	5 (35.7)	0 (0.0)	< 0.01
Respiratory Failure Requiring IMV, n (%)	3 (21.4)	0 (0.0)	0.06
Respiratory Failure Requiring NIV, n (%)	2 (14.3)	0 (0.0)	0.153
ICU Length of Stay (days) ^†^	4 (4, 7)	2 (1, 3)	< 0.001
Hospital Length of Stay (days)^†^	9 (7, 13)	4 (4, 8)	< 0.001

† Values are median with 25 and 75 percentiles in parenthesis.

Definition of abbreviations: ICU, intensive care unit; IMV, invasive mechanical ventilation; NIV, noninvasive mechanical ventilation.

**FIGURE 1:**
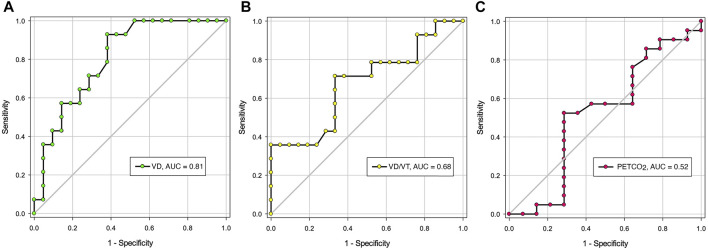
The area under the Receiver Operating Characteristic Curves for resting VD, VD/VT and PETCO_2_. **(A)**. Area under the curve (AUC) for VD: 0.81 (*p* = 002); **(B)**. AUC for VD/VT: 0.68 (*p* = 0.077); **(C)**. AUC for PETCO_2_: 0.52 (*p* = 0.839). Definition of abbreviations: VD, dead space volume; VD/VT, dead space to tidal volume ratio; PETCO_2_, end-tidal CO_2_ pressure. NS, not significant.

Figure 1 shows the ROC analysis for VD, VD/VT and PETCO_2_. The area under the ROC curve (AUC) was statistically significant for VD only (0.81, *p* = 0.002). The AUC for VD/VT and PETCO_2_ was 0.68 (*p* = 0.077) and 0.53 (*p* = 0.839), respectively. A threshold value for VD at or below 0.229 L appears to be an acceptable predictor for the lack of PPC with sensitivity of 62% (95% Confidence Interval (CI): 38%–82%), and specificity of 93% (95% CI: 66%–100%). The positive and negative likelihood ratio was 8.7 and 0.4, respectively.

## Discussion

In this pilot study, our major findings were, 1) the preoperatively measured resting physiological dead space volume (VD) was the only variable separating groups of patients with and without PPC; 2) resting VD was also a significant predictor of PPC after robotic-assisted lung cancer resection surgery with a threshold value of 0.229 L; 3) resting end-tidal PCO_2_ was not useful in predicting the frequency of PPC; and 4) the PPC following robotic-assisted lung resection surgery was relatively high of 40%.

### Resting Physiological Dead Space

Resting VD, the sum of anatomical and alveolar dead space, was obtained from ventilatory efficiency ( $\dot{V}$ E/ $\dot{V}$ CO2) via breath-by-breath gas analysis and arterial PCO_2_ rather than PETCO_2_, and therefore, avoiding inaccurate estimate (Lewis et al., 1994). The elevated resting VD in the PPC group might be accounted for by an increase in ventilation-perfusion inequalities despite similar spirometry and DLCO indices (Sinha et al., 2011; Robertson, 2015). A crude estimate of ventilation-perfusion inequalities, PaCO_2_-PETCO_2_ difference was similar in both groups (Table 3). However, on a closer observation there were subtle differences in the measures of airflow limitation (FEV_1_/FVC, FEV_1_ and FVC) between those with and without PPC; with a higher prevalence of patients with COPD in the PPC group (79% vs. 52% without PPC). The underlying COPD cannot be dismissed to account for the high VD in the PPC group (Table 1).

While VD differed significantly between groups with and without PPC, VD/VT did not. The lack of significant difference in VD/VT was possibly related to the mildly elevated VT in the PPC group despite statistically insignificant (Table 3). Patients with a high resting VD may require higher compensatory VT to achieve effective ventilation and removal of CO_2_. Thus, the VD/VT ratio was similar for both groups with and without PPC. There have been several studies in healthy subjects describing this phenomenon of elevated VT in response to elevated VD during exercise (Wasserman et al., 1967), and at rest (Krishnan et al., 1997). The possible mechanisms may include alterations in the PCO_2_ time profile or oscillations sensed by airway and/or pulmonary receptors, carotid chemoreceptors, or the central chemoreceptors (McParland et al., 1991). However, studies have shown that airway receptors (Krishnan et al., 1997) and carotid chemoreceptors (Syabbalo et al., 1993) do not play a major role in the mechanism of increased VT in response to elevated VD. Central chemoreceptors may possibly have a predominant role.

As shown in Table 2, a large percentage of our patients with PPC had mild COPD (prebronchodilator FEV_1_/FVC of 65.8%) with an average resting VD/VT of 0.35. Our findings were comparable to that of Elbehairy et al. (2015) with their mild COPD patients. The average prebronchodilator FEV_1_/FVC was 59.5% and resting VD/VT of 0.37. VT and frequency were not reported; however, minute ventilation ( $\dot{V}$ E) was lower, 11.8 L/min compared to 17.4 L/min in our patients. The lack of increased $\dot{V}$ E in their study was not apparent, however, altered breathing pattern with ventilatory constraint such as low VT and high frequency would increase VD/VT (Whipp, 2006; Smith and Olson, 2019).

In this trial or experimental cohort, resting VD appears a useful predictor of PPC following lung resection surgery at a threshold value of 0.229 L, with area under ROC curve of 0.81 (Figure 1). This confers the advantage for those patients who cannot or decline to perform CPET in the preoperative evaluation of postoperative complications risk after lung resection surgery. The positive likelihood ratio was 8.7, suggesting that a positive result is 8.7 times as likely for a patient who experienced PPC as one who did not. At the above threshold value, we obtained a sensitivity of 62% and specificity of 93%. The Youden index is 55% [(62% + 93%)−100%] suggesting that the resting VD threshold value yields an appreciable fraction that may be misclassified (Bewick et al., 2004). For this reason, this threshold value needs to be prospectively validated in a large number of patients.

### Resting End-Tidal PCO_2_

A recent study of Brat et al. (2016) demonstrated that resting PETCO_2_ was a strong predictor of PPC following lung resection surgery. In contrast, our results differed in that resting PETCO_2_ was similar for both groups with and without PPC (Table 3). Differences might be related to patient characteristics such as 36% of their cohort were female; and/or variability in PETCO_2_ estimate. PETCO_2_ is critically influenced by breath-by-breath changes in the pattern of breathing. It is determined by the timing of end-exhalation during the alveolar phase-III of CO_2_ of each breath. In patients with airflow limitation a plateau of alveolar phase-III could not be attained resulting in large variability of its measurement and a large gradient between PaCO_2_ and PETCO_2_ (Sinha et al., 2011). Furthermore, particularly in COPD patients, wasted ventilation contaminates the measurements, as inhaled CO_2_ that doesn’t take part in gas-exchange is exhaled and dilutes the mixed expired CO_2_. Our study did not show significant difference in PaCO_2_-PETCO_2_ between both groups, probably related to variability of PETCO_2_.

### Postoperative Pulmonary Complications Following Robotic-Assisted Lung Surgery

CPET has been considered as the gold standard in the evaluation of PPC following lung resection (Brunelli et al., 2013). Interestingly, neither Peak $\dot{V}$ O_2_ nor $\dot{V}$ E/ $\overset{˙}{V}$ CO_2_ slope were able to preoperatively distinguish patients with and without PPC. Peak $\dot{V}$ O_2_ for both groups averaged 20 L/min/Kg and $\dot{V}$ E/ $\dot{V}$ CO_2_ averaged less than 34 (Table 3). Most likely this was due to our patients’ absence of, or mild airflow limitation; or compensated stable heart failure. The PPC rate in our cohort was considered relatively high of 40%. However, this complication rate in the older patients as in our cohort, were within the range of others, 33%–44% (Velez-Cubian, et al., 2015; Veluswamy et al., 2020).

### Study Strength and Limitation

The strength of our study is the prospective design of the study and the use of PaCO_2_ rather than PETCO_2_ in the calculation of VD. However, our study has several shortcomings. First, our population consists of only male gender from a single institution, the Veterans Affairs Healthcare System. The results of this study, therefore, may not be applicable to the female population and a multi-center study would be desirable. Second, although the number of subjects studied exceeded the calculated sample size, the present study is relatively small involving a trial cohort and will require a validation cohort to test whether resting VD will hold as a reliable predictor of PPC risk. Furthermore, a multivariate analysis to assess the confounding effects of COPD cannot be performed. Nonetheless, resting VD stood out as a potentially useful predictor of PPC risk after robotic-assisted lung surgery. Third, all lung resections involved lobectomy except for two segmentectomies, and no pneumectomy was performed. Hence, resting VD as predictor of PPC is only applicable in those patients with lobectomies.

### Conclusion

In summary, our prospective study demonstrated that following robotic-assisted lung resection for suspected or biopsy-proven lung cancer, resting VD obtained preoperatively separated those patients with and without postoperative pulmonary complications. Resting VD is also a potential predictor for postoperative pulmonary complications risk. However, this will have to be further validated with a large number of patients.

## Data Availability

The original contributions presented in the study are included in the article, further inquiries can be directed to the corresponding author.
